# Alcoholic Liver Disease and Systemic Inflammatory Response Syndrome: Mortality Prediction Using Biomarkers and Clinical Scores

**DOI:** 10.3390/jcm14217580

**Published:** 2025-10-25

**Authors:** Tijana Glisic, Bojan Korica, Milica Stojkovic Lalosevic, Nevena Baljosevic, Jasna El Mezeni, Marko Kartal, Dusan Dj Popovic, Jelena Martinov Nestorov, Snezana Lukic, Dragana Mijac

**Affiliations:** 1 Clinic of Gastroenterology and Hepatology, University Clinical Center of Serbia, 11000 Belgrade, Serbia; koricabojan@gmail.com (B.K.); jasnavujisic@gmail.com (J.E.M.); jelenamartinov@yahoo.com (J.M.N.); lukic.snezana@gmail.com (S.L.); draganamijac@gmail.com (D.M.); 2 Faculty of Medicine, University of Belgrade, 11000 Belgrade, Serbia; dr.dusan.popovic@gmail.com; 3 Emergency Center, University Clinical Center of Serbia, 11000 Belgrade, Serbia; baljosevic.nevena@yahoo.com (N.B.); markocl.kartal@gmail.com (M.K.); 4 Department of Gastroenterology, Clinical and Hospital Center “Dr Dragisa Misovic-Dedinje”, 11000 Belgrade, Serbia

**Keywords:** cirrhosis, systemic inflammatory response syndrome, non-invasive scores, laboratory biomarkers

## Abstract

**Background/Objectives**: Cirrhosis is an irreversible state of chronic liver disease. Systemic inflammatory response syndrome (SIRS) is a severe complication and significantly contributes to lethal outcomes in cirrhotic patients. We studied a group of cirrhotic patients with SIRS admitted to our centre, assessing the relationship with in-hospital outcomes. **Methods**: The study population included 102 patients with alcoholic cirrhosis and SIRS. Laboratory biomarkers, the model for end-stage liver disease, the model for end-stage liver disease—natrium, the Acute Physiology and Chronic Health Evaluation II score, CLIF-C organ failure, the systemic immune-inflammation index score (S II), and the Cirrhosis Acute Gastrointestinal Bleeding (CAGIB) score were tested in relation to the mortality risk using receiver operating characteristic (ROC) curves. **Results**: Our results demonstrated that values of sodium, chlorides, and albumin significantly correlated with 7-day survival. The area under the curve’s (AUCs) values for sodium, chlorides, and albumin were 0.542, 0.627, and 0.610, respectively, for 7-day mortality prediction. The CAGIB score significantly correlated with 7-day mortality, with the cut-off value of −7.86 (AUC: 0.674, 95% CI (0.555–0.794)). For the assessment of 28-day mortality, the AUC values for sodium, chlorides, and albumin were 0.630, 0.654, and 0.661, respectively. Additionally, the cut-off value of the CAGIB score was found to be −7.84 (AUC: 0.625, 95% CI (0.509–0.740)) in 28-day mortality prediction. **Conclusions**: Sodium, chlorides, albumin, and the CAGIB score are reliable predictors of 7-day and 28-day in-hospital mortality in patients with advanced alcoholic liver disease and SIRS.

## 1. Introduction

Cirrhosis, as the final stage of chronic liver disease, is associated with significant immunological impairment [1]. Patients with liver cirrhosis are considered immunocompromised due to their compromised ability to defend against microorganisms, including bacteria, making these patients more prone to infections, which further increase morbidity, mortality, and progression with acute-on-chronic liver failure (ACLF) [2]. Globally, the incidence of liver cirrhosis shows significant geographic and temporal variations. The liver cirrhosis incidence reached 58.4 million in 2021, up from 36.9 million in 1990. The absolute number of deaths from alcoholic liver disease has increased, particularly in parts of Eastern Europe and Latin America [3,4]. From 1990 to 2019, the primary causes of liver cirrhosis were hepatitis B and hepatitis C, but in the last years, alcohol-related cirrhosis has increased significantly.

Systemic inflammatory response syndrome (SIRS) represents one of the most important features of sepsis and persistent inflammation. This generalised condition is characterised by an immune response to microorganisms or aseptic inflammation, which can lead to a disturbed microcirculation, visceral hypoperfusion, and multi-organ failure [5]. SIRS has frequently been observed in patients with advanced chronic liver disease. Although there is an evident increase in the number of cases of chronic liver failure worldwide, certain conditions, such as hepatic encephalopathy, hypersplenism, or the use of beta-blockers, can mask the presence of SIRS, making the criteria for diagnosing the infection unreliable.

At the end of the last century, the SIRS criteria served as an important prognostic tool for the clinical assessment of patients with infection. These criteria include tachycardia, tachypnoea, leukocyte disturbance (leucocytosis or leukopenia), and an altered body temperature (either hyper or hypothermia) [6]. SIRS is among the most serious complications observed in patients with liver cirrhosis, irrespective of the underlying cause, and significantly contributes to further liver function worsening, negatively altering the course of the disease [7,8,9,10]. There is some evidence that systemic inflammation enhances the risk for complications such as variceal haemorrhage, hepatic encephalopathy, and ACLF [11,12].

The development of ACLF is most commonly initiated by bacterial infections (BIs). SIRS occurs in 57–70% of patients with liver cirrhosis, and it is well known that bacterial infections can lead to this condition [13]. The predictive value of SIRS criteria in patients with advanced chronic liver disease, for diagnosing BI, is insufficient [14,15]. Some inflammatory markers have demonstrated superior value in the diagnosis of bacterial infections in cirrhosis [16]. In this context, the previous study of Papp et al. has shown a correlation between C-reactive protein levels and the progression of sepsis [16].

Given that patients with decompensated cirrhosis often require hospitalisation in intensive care units (ICUs), a significant number of these patients face an unfavourable prognosis, with mortality rates of 33% in the ICU and an overall in-hospital mortality of nearly 48%. Regarding the ACLF grade and scores for organ failure, patients with ACLF in the ICU have high mortality rates—from 36% to 89% [17,18]. Patient outcomes are largely determined by the severity of hepatic and extrahepatic organ failure [19].

Given the complexity of infection development and management in patients with liver cirrhosis, the use of inflammatory biomarkers and prognostic scores as non-invasive predictors of mortality appears to be highly valuable.

Prognostic scores are reliable, cheap, and fast in this decision-making and have been used often in outcome prediction in patients with progressive chronic liver disease. The initial evaluation of critically ill patients with advanced chronic liver disease is the first measure required in the stratification, and SIRS criteria have often been used for that purpose. However, due to their poor discriminatory value, the use of SIRS is no longer recommended [20]. There are some prognostic scores specific for liver cirrhosis like Child–Pugh (CP) [21], the Model for End-stage Liver Disease (MELD) [22], and the Chronic Liver Failure-Consortium ACLF (CLIF-ACLF) [23] score, for example, or the Sequential Organ Failure Assessment (SOFA) [24] score and the Acute Physiology and Chronic Health Evaluation II (APACHE II) [25] score, which we use for all patients admitted to the ICU. One of the newer scores in use is the CAGIB score [26]. Initially, this score was developed and validated to assess the nosocomial mortality rate in patients with GI bleeding and advanced chronic liver disease. Previous studies have not addressed the performance of this score in complicated conditions in patients with advanced chronic liver disease, such as patients who have been diagnosed with SIRS and ACLF. To limit the use of upper endoscopy, in our study, we used the EVendo score [27] as a non-invasive tool in assessing the presence of esophagogastric varices. Although patients had previously undergone endoscopy to determine the presence of varices, we wanted to verify the utility of this score in our patient group.

The primary objective of this retrospective study was to evaluate laboratory biomarkers and non-invasive scoring systems in patients with SIRS and ACLF, with the aim of identifying which scores demonstrated the largest diagnostic accuracy in mortality prediction and need for treatment in an intensive care unit.

## 2. Materials and Methods

In this retrospective analysis, we assessed patients with ACLF and SIRS who were admitted to the Emergency Centre at the University Clinical Centre of Serbia. Our study included 102 patients with alcoholic advanced chronic liver disease who were admitted to our emergency department between 2020 and 2025. Patients diagnosed with advanced chronic liver disease based on relevant clinical information, imaging methods (ultrasonography and computerised tomography of liver and other organs), laboratory findings, and/or histological reports were included in this research. Subjects in the following situations were excluded: (1) minor patients; (2) patients with malignancy; (3) previous surgery of liver or spleen; (4) previous transjugular intrahepatic portosystemic shunt; (5) thrombosis of the portal venous system; (6) use of immunosuppressive drugs; and (7) human immunodeficiency virus (HIV) infection.

All enrolled patients were diagnosed with advanced alcoholic chronic liver disease.

The diagnosis of acute-on-chronic liver failure (ACLF) was in accordance with the European Association for the Study of the Liver–Chronic Liver Failure Consortium (EASL-CLIF) [28]. Liver failure is defined as the level of serum bilirubin above or equal to 85 mmol/L, and coagulation failure is considered an international normalised ratio ≥ 1.5. Those are mandatory parameters to estimate liver dysfunction. Hepatic encephalopathy (HE) is diagnosed by a physical examination and the neurological status and is an important feature of liver failure assessment. Patients with HE were classified using the guidelines of the American Association for the Study of Liver Diseases and the European Association for the Study of Liver Practice, Subcommittee on Hepatic Encephalopathy [29].

All patients with decompensated advanced chronic liver disease admitted to our intensive care unit were further evaluated for SIRS according to the following criteria: (1) body temperature > 38 °C or <36 °C, (2) respiratory rate ≥ 20 beats/min, (3) heart rate ≥ 90 beats/min, and (4) white blood cell (WBC) count ≥ 12,000/mm^3^ or ≤4000/mm^3^. The presence of two or more criteria in patients with advanced chronic liver disease included them in this study.

This study was in accordance with the regulations of the Ethical Committee of the University Clinical Centre of Serbia (approval number: 772/8; approval date: 27 March 2025). This study was performed in line with the principles of the Declaration of Helsinki (1989). Each patient or patient relatives (in the case of an altered mental state) signed written consent for participation in this study.

### 2.1. Data Collection

The data used in this research were collected from the medical records of patients: age; sex; physical examination finding such as ascites, hepatic encephalopathy (HE), splenomegaly, active variceal bleeding; personal history data: previous hospitalisations for liver disease, comorbidities (arterial hypertension, diabetes, chronic heart diseases, pulmonary diseases); laboratory findings: red blood cells (RBC), haemoglobin (Hb), white blood cells (WBC), neutrophils (Ne), lymphocytes (Ly), monocytes (Mo), eosinophils (Eo), platelets (PLT), alanine aminotransferase (ALT), aspartate aminotransferase (AST), alkaline phosphatase (ALP), γ-glutamine transferase (GGT), lactate dehydrogenase (LDH), international normalised ratio (INR), fibrinogen, albumin (ALB), total bilirubin (TBIL), blood urea nitrogen (BUN), creatinine (Cr), glucose, sodium (Na), potassium (K), chlorides (Cl), C-reactive protein (CRP), procalcitonin (pct). Additionally, we calculated the model for end-stage liver disease (MELD) [22], model for end-stage liver disease—natrium (MELD-Na) [30], neutrophil-to-lymphocyte ratio (NLR) [31], derived neutrophil-to-lymphocyte ratio (dNLR) [32], platelet-to-lymphocyte ratio (PLR) [33], systemic immune-inflammation index score (S II) [34], Acute Physiology and Chronic Health Evaluation II score (APACHE II) [25], and Cirrhosis Acute Gastrointestinal Bleeding (CAGIB) score [26].

To diagnose acute-on-chronic liver failure (ACLF) in patients with acute decompensation of cirrhosis, we used the CLIF-C organ failure (CLIF-C OF) score. It helps assess the severity of ACLF through the evaluation of six organ systems (liver, kidney, brain, coagulation, circulation, and respiration) [35]. The CLIF-C acute-on-chronic liver failure (ACLF) score is used to predict the prognoses of patients with ACLF. Accordingly, four groups of patients with acutely decompensated cirrhosis are defined: one group of patients without ACLF and three groups of patients with an increasing severity of ACLF (grade 1, grade 2, and grade 3) on the basis of the type and number of OF(s) [28].

Patients were monitored daily due to critical illness, requiring prolonged ICU treatment.

All scores are presented in Table 1.

### 2.2. Evaluation of Infection and Septic Shock

Each patient underwent a blood culture, urine culture, and tracheal aspirate, if intubated. Bloodstream infection was defined as bacterial growth in blood cultures of microbiologic species without contamination. Septic shock was defined as severe sepsis with hypotension despite intensive fluid resuscitation.

### 2.3. Statistical Analysis

Statistical analyses were performed using SPSS software version 20.0 (IBM, Chicago, IL, USA). Descriptive statistics were used to summarise demographic and clinical data, expressed as means ± standard deviations, medians with interquartile ranges (IQRs), or percentages, as appropriate. The Kolmogorov–Smirnov test was applied to assess the normality of data distribution. Differences between groups were evaluated using Student’s t-test, the Mann–Whitney U test, or the chi-square test.

Prior to ROC curve analyses, preliminary correlation analyses (Pearson’s for normally distributed variables and Spearman’s for non-parametric variables) were performed to identify laboratory and clinical parameters that were significantly associated with 7-day and 28-day mortality. Only variables showing a significant correlation (*p* < 0.05) were subsequently included in the ROC curve analyses to improve clarity and reduce model redundancy.

Sensitivity, specificity, and optimal cut-off values were determined using ROC curves and Youden’s index. Logistic regression and Cox proportional hazards analyses were conducted at the univariable level to explore associations between predictors and mortality outcomes, given the limited sample size and to avoid model overfitting. Survival analysis was performed using the Kaplan–Meier method, and group differences were assessed with the log-rank test. A *p*-value less than 0.05 was considered statistically significant.

Given the exploratory nature of this study and the limited number of events, formal calibration (Hosmer–Lemeshow test) and reclassification metrics (Net Reclassification Improvement, NRI) were not performed. These analyses will be considered in future validation studies with larger patient cohorts.

## 3. Results

### 3.1. Demographic Characteristics

Our study population included 102 patients with advanced alcohol chronic liver disease who met the criteria for SIRS. Demographic and laboratory data for the study population are presented in Table 2. The mean age of our patients was 56.6 ± 13.86 years, and 70.6% were male. Among our patients, active variceal bleeding was observed in 18.6% of cases, peritoneal free fluid (ascites) in 81.4%, and hepatic encephalopathy in 70.6%. In our study group, 30.4% of patients survived and 69.6% of them did not. There was no statistically significant difference in survival rates between the genders in the study population.

We found significantly different values of white blood cells (WBC), neutrophils, monocytes, INR, albumin, creatinine, sodium, chlorides, lactate dehydrogenase, mean arterial pressure (MAP), MELD, MELD-Na, APACHE II, and CAGIB score between the 7-day survived and non-survived patients’ group (Table 3), while for the 28-day mortality, WBC, neutrophils, monocytes, INR, albumin, creatinine, sodium, chlorides, lactate dehydrogenase, CRP, mean arterial pressure (MAP), blood oxygen saturation, MELD, MELD-Na, APACHE II, and CAGIB score showed significantly different values between the examined groups (Table 4).

In the current study, we found that heart disease (chronic heart failure) increased the risk of 7-day mortality by more than 7.8-fold (Table 5), and GI bleeding was associated with a 2.54-fold greater risk of a 7-day lethal outcome in our study group (Table 6). Table 5 and Table 6 present the results of univariable Cox regression analyses examining the association between heart disease, gastrointestinal bleeding, and 7-day mortality.

### 3.2. Prognostic Factors for 7-Day Lethal Outcome in Cirrhotic Patients with SIRS

**Figure 1:**
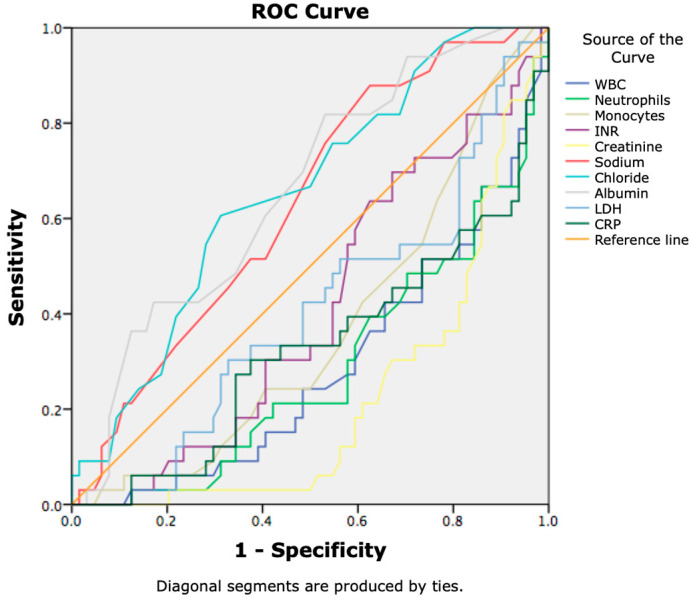
Receiver operating characteristic (ROC) curves showing the diagnostic performance of laboratory biomarkers for identifying 7-day mortality in patients with SIRS and cirrhosis.

The ROC curve of variables for 7-day mortality is presented in Figure 1. From the analysis of the prediction of a 7-day lethal outcome, the AUCs of sodium, chlorides, and SIRS were 0.542, 0.627, and 0.610, respectively. This analysis for 7-day mortality showed a sensitivity of 72.7% and specificity of 33.3% for the cut-off value of 132.5 for sodium, sensitivity of 76% and specificity of 42% for the cut-off value of 95.5 for chlorides, and sensitivity of 73% and specificity of 39% for the cut-off value of 25.5 for albumin, respectively.

**Figure 2:**
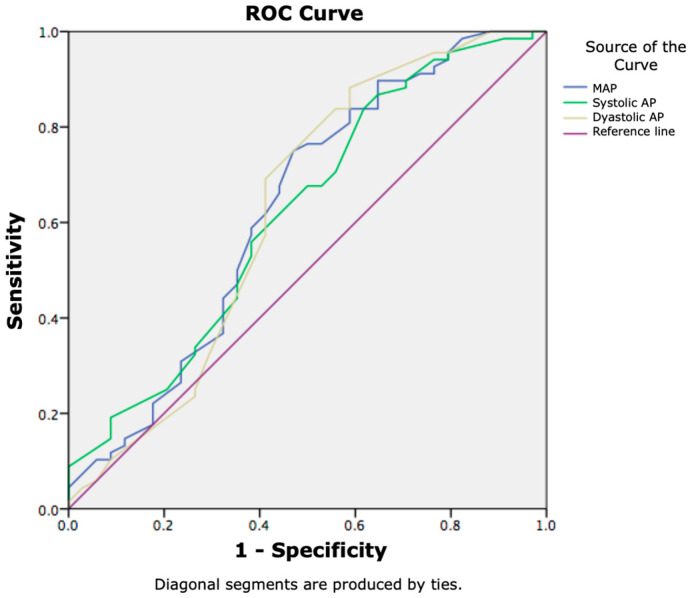
Arterial blood pressure in predicting 7-day mortality in patients with SIRS and liver cirrhosis.

Furthermore, mean arterial blood pressure was significantly correlated with a lethal outcome in the population in this study (*p* < 0.01). The ability to use the examined vital signs to predict the 7-day mortality was tested using ROC curves. Mean blood pressure had the discriminative ability to predict 7-day survival with a sensitivity of 75% and specificity of 53% for the cut-off value of 75.5 (AUC: 0.630, 95%CI (0.507–0.753)) (Figure 2).

**Figure 3:**
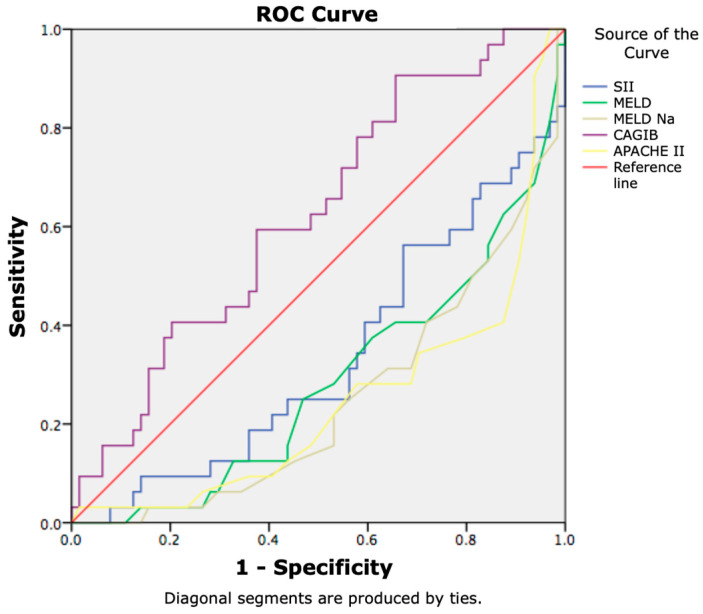
Clinical scores in prediction of 7-day mortality.

In addition, we examined the correlation between non-invasive scores and short-term mortality in our study population. Among the all examined scores, only the CAGIB score statistically significant correlated with 7 day-mortality, with a sensitivity of 75% and specificity of 56% for the cut-off value of −7.86 (AUC: 0.674, 95% CI (0.555–0.794)) (Figure 3).

**Figure 4:**
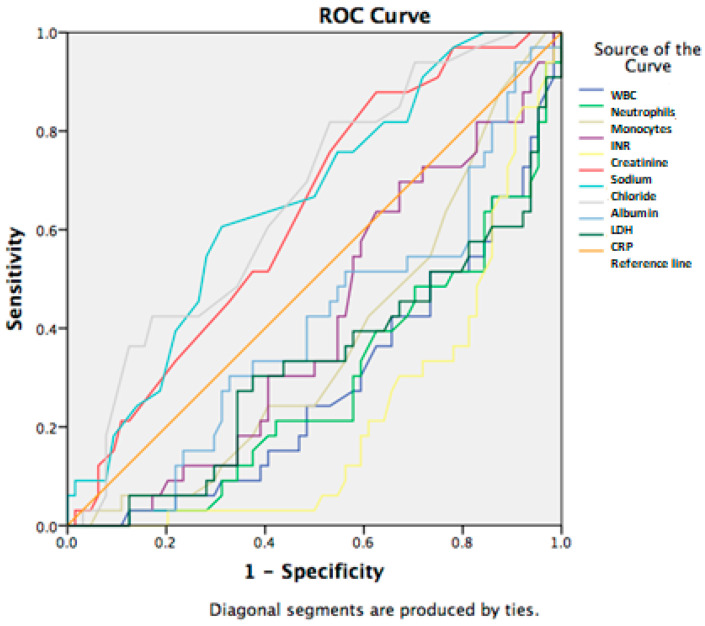
Laboratory biomarkers in prediction of 28-day mortality in patients with SIRS and cirrhosis.

In the analysis of the laboratory biomarkers and blood pressure in regard to the 28-day mortality, sodium, chlorides, and albumin were significantly associated with poor outcomes, as well as systolic, diastolic, and mean blood pressure. However, the AUROC curve results generated were all satisfactory. Sodium, chlorides, and albumin had similar AUCs of 0.630, 0.654, and 0.661, with the cut-off value of 133.5 for sodium, the cut-off value of 97.5 for chlorides, and the cut-off value of 27.5 for albumin (Figure 4).

**Figure 5:**
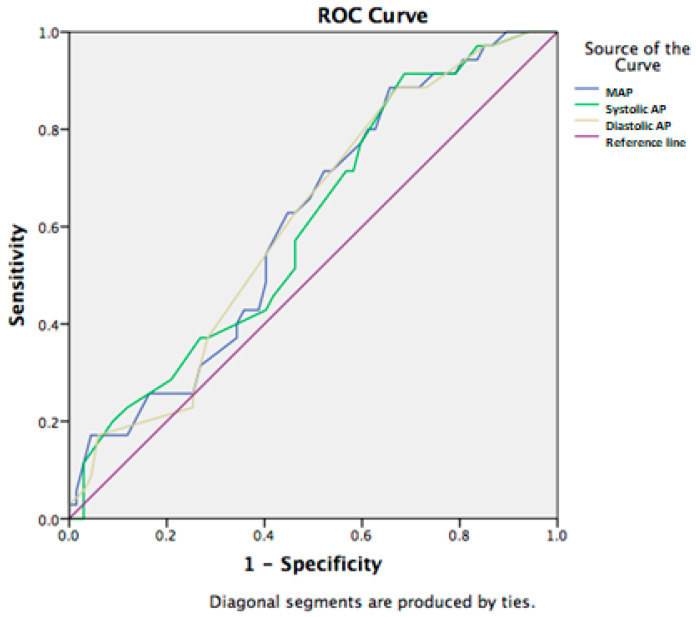
Prediction of 28-day mortality in patients with liver cirrhosis and SIRS using arterial blood pressure.

Mean blood pressure statistically significantly correlated with 28-day mortality, and using the ROC curve, we calculated the mean blood pressure with the cut-off value of 75.5, with a sensitivity of 77% and specificity of 40% (AUC: 0.609, 95% CI (0.498–0.721) (Figure 5).

**Figure 6:**
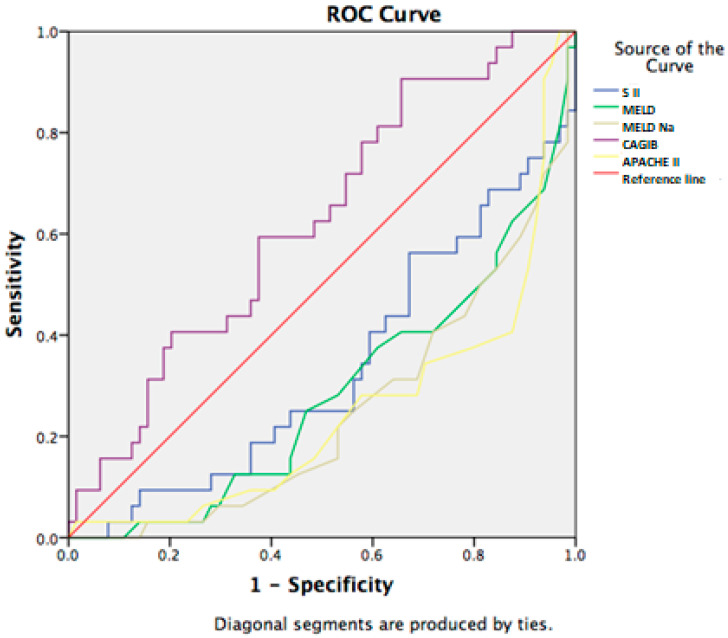
Non-invasive scores in prediction of 28-day mortality.

Additionally, we investigated the diagnostic accuracy of MELD, MELD-Na, APACHE II, SII, and the CAGIB score for the 28-day mortality in patients with advanced chronic liver disease and SIRS. The cut-off value of the CAGIB score was found to be −7.84 (AUC: 0.625, 95% CI (0.509–0.740)), which had a sensitivity of 72% and a specificity of 43% (*p* = 0.04; *p* < 0.05). A ROC curve has been provided in Figure 6.

### 3.3. Kaplan–Meier Survival Analysis

**Figure 7:**
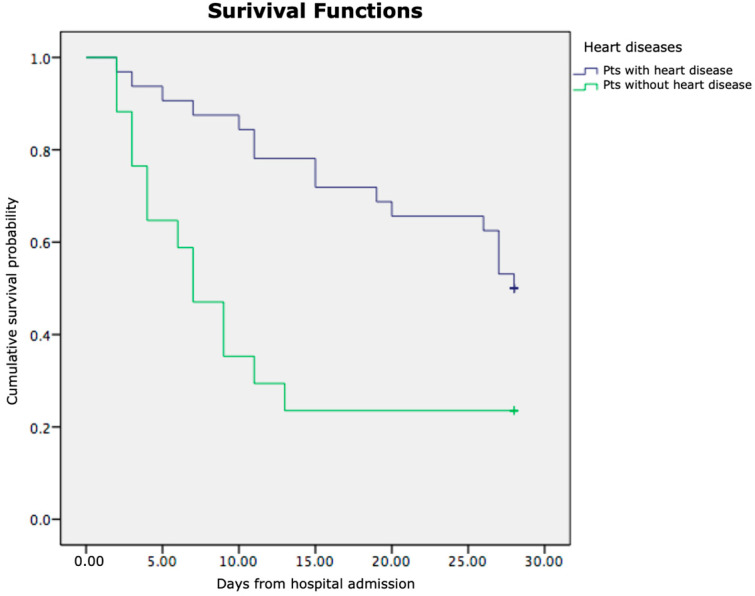
Kaplan–Meier survival curves showing cumulative survival probability in patients with and without heart disease.

**Figure 8:**
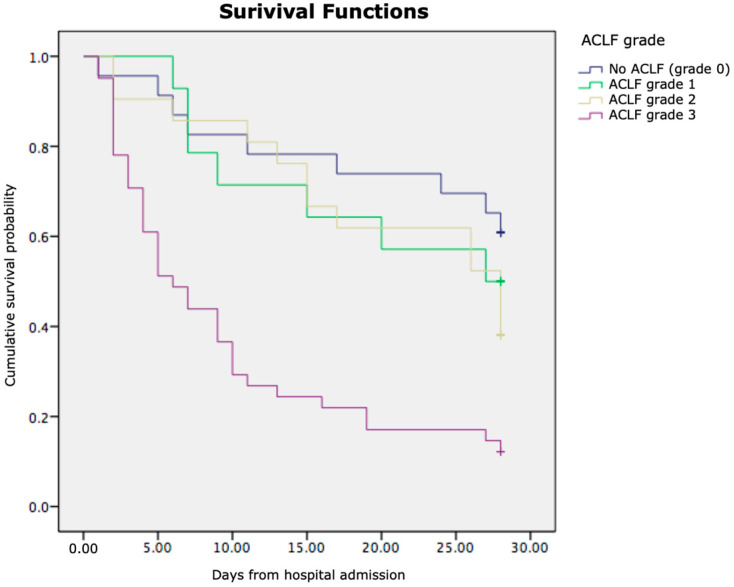
Kaplan–Meier survival curves showing cumulative survival probability according to ACLF grade (Grade 0, Grade 1, Grade 2, Grade 3).

Kaplan–Meier survival analysis demonstrated significant differences in cumulative survival among the examined patient subgroups. As shown in Figure 7, patients with pre-existing heart disease had markedly lower cumulative survival compared to those without heart disease (log-rank *p* < 0.05). Similarly, Figure 8 illustrates that patients with higher grades of acute-on-chronic liver failure (ACLF Grades 2–3) exhibited significantly poorer survival than those with lower grades (ACLF Grades 0–1) (log-rank *p* < 0.01).

In both figures, the *x*-axis represents the time in days from hospital admission, and the *y*-axis represents the cumulative survival probability. These findings indicate that the presence of cardiac comorbidity and a greater ACLF severity are independently associated with reduced short-term survival in patients with liver cirrhosis and SIRS.

## 4. Discussion

Liver cirrhosis is characterised by a systemic inflammatory and hyperdynamic hemodynamic state that can mimic signs of infection. In an attempt to find new, more accurate criteria for detecting early signs of infection, the SIRS criteria are still used. In this group of patients, mortality is extremely high, and prognostic scores can help in making decisions about further therapeutic approaches.

Our analysis revealed that most patients in the study population had alcoholic liver cirrhosis, consistent with the findings of Michelena et al. [36]. Their research highlighted the central role of SIRS in contributing to multi-organ failure and increased mortality among individuals with alcoholic hepatitis. This association may be attributed to enhanced intestinal bacterial translocation, driven by alcohol-induced increases in gut permeability.

In our study, we found significant differences in levels of white blood cells (WBC), neutrophils, monocytes, INR, albumin, creatinine, sodium, chlorides, lactate dehydrogenase, mean arterial pressure (MAP), MELD, MELD-Na, APACHE II, and CAGIB score between 7-day survivors and non-survivors. Emerging evidence indicates that elevated leukocyte counts could be associated with an increased short-term mortality risk in individuals with liver failure [37]. It has been suggested that neutrophils have a significant role in early phases of the inflammatory cascade, caused by tissue damage [38]. Moreover, it has been found that there is an important relationship between monocyte deactivation and adverse outcomes in patients with sepsis [39]. Chung et al. found, in patients who had normal blood cell counts before the disease course, that monocyte counts were increased in those who survived, while they were decreased in those who had a lethal outcome, from the premorbid period to sepsis [40]. The discrepancy between our results and previous studies may be explained by the distinct immunological profile of cirrhosis, which involves both a heightened pro-inflammatory response and concurrent immune dysfunction. Despite elevated monocyte and neutrophil counts, their reduced functional capacity likely contributes to poorer clinical outcomes.

Electrolyte imbalance, especially hyponatraemia, has been associated with poor outcome in patients with advanced chronic liver disease. There is significant amount of data indicating that hypochloraemia enables better information relative to the outcome in these patients. The pathophysiological mechanisms underlying the association between low serum chloride levels and poor outcomes in chronic liver disease appear to involve complex interactions between electrolyte imbalance, renal function, and systemic haemodynamic [41]. Notably, our data confirmed these findings. Portal hypertension and splanchnic vasodilation may contribute to hypochloraemia in advanced cirrhosis patients. These two conditions cause arterial hypovolaemia and subsequent activation of endogenous vasoconstrictor systems, which lead to disproportionate water retention. At the end, it results in dilutional hypochloraemia in addition to hyponatraemia. Other factors that contribute to hypochloraemia are adrenal insufficiency, malnutrition, salt-restricted diets, and concomitant heart failure [42].

Prognostic scores such as MELD, MELD-Na, and APACHE are known to show a statistically significant difference in values between the groups of survivors and non-survivors with advanced liver disease [43,44], which is similar to our results, although we did not find data in the available literature that processed these prognostic scores in the group of patients with advanced liver disease and SIRS. When examining the performance of predictive scores, Ahmed et al. found that the APACHE score was superior in predicting short-term mortality [45]. A prognostic score that has not been applied to the population of patients with SIRS and ACLF, but which showed significant potential in predicting mortality in our study group was the CAGIB score. Although it was initially applied in the group of patients with cirrhosis and acute GI bleeding, the components of this score do not make it specific only to GI bleeding, which is why we incorporated it into our research.

When analysing the significance of the presence of heart disease, our results showed that there was a statistically significant difference in survival for patients who had heart disease compared to those who did not. Yazdanyar et al. showed that patients with LC who are hospitalised for decompensated heart failure have a higher mortality risk and decreased likelihood of discharge by the targeted length of stay [46].

The event that significantly correlated with 7-day mortality in our patient group was GI bleeding. Our results showed that GI bleeding increased the risk of a lethal outcome by 2.54-fold. Other studies also presented similar results [47].

In the analysis of the association of electrolytes and albumin in the prediction of 7-day and 28-day mortality, we found that there was a significant association of sodium, chlorides, and albumin with lethal outcomes. Studies have shown that hyponatraemia is a significant predictor of mortality in liver cirrhosis patients [48]. Furthermore, the serum sodium value has been incorporated into the new version of the MELD score, currently the most widely used prognostic score in patients with advanced chronic liver disease [49]. In our investigation, hypochloraemia showed the highest association with lethal outcomes in patients with LC and SIRS, even compared to hyponatraemia, which agrees with various studies. The study by Semmler et al. showed that serum chloride, not sodium, had a significant association with mortality in an intensive care unit (ICU) cohort of cirrhotic patients [50]. The study conducted by Ji and Li compared a group of critically ill patients with cirrhosis who had hypochloraemia versus a non-hypochloraemic group, and they found that hypochloraemic patients had higher mortality in the ICU. Moreover, every unit decrease in chloride level predicted a 6% increase in mortality [51].

Mean arterial pressure (MAP) is a reliable indicator of circulatory dysfunction in patients with liver cirrhosis [52]. The present study showed that the patients who died after 7 or 28 days had lower MAP values than those who survived. The results indicated that the baseline MAP level was an important independent predictor of death in our group of cirrhotic patients with SIRS. Also, we analysed the risk of different cut-off points of MAP in all the patients to confirm the best cut-off point. Our results revealed a notable increase in death when MAP was under 75.5 mmHg in patients with cirrhosis and SIRS. These results are in accordance with other studies [53], although our cut-off values are lower, possibly due to the presence of SIRS. Also, in our group of patients, there were those with septic shock, which had a statistically significant impact on 7-day survival.

The diagnostic accuracy of MELD, MELD-Na, APACHE, and the SII score for the 7-day and 28-day mortality in cirrhotic patients with SIRS was not very high in our study, but it is interesting that the CAGIB score showed the best performance in predicting short-term mortality. Previous studies have examined the relationship between the CAGIB score and outcomes in patients with cirrhosis and acute GI bleeding [54]. Our study aimed to investigate the potential capacity of this new score in the group of the most severe patients with cirrhosis and SIRS. Given that the laboratory variables that make up this score are not narrowly specific to GI bleeding alone, further research is needed to determine the potential for the application of the CAGIB score in predicting various complications and outcomes in patients with liver cirrhosis.

When we analysed the prediction of a lethal outcome in more detail in relation to the exact time of in-hospital mortality from hospital admission, Kaplan–Meier analysis indicated significantly reduced survival in patients with heart disease as a comorbidity, as well as in patients with a high ACLF grade. Heart failure (HF) and liver disease often coexist, and in states of advanced liver disease, deterioration of these patients occurs significantly more rapidly, because of systemic disorders and diseases that affect both organ systems. HF may lead to liver disease, but liver disease per se may cause heart failure in the absence of other cardiovascular abnormalities [55,56]. The unfavourable outcomes observed in patients with cirrhosis, SIRS, and concurrent heart failure are likely due to a complex interplay of factors. Heart failure may hasten deterioration by contributing to hypoxaemia and necessitating inotropic support to address inadequate tissue perfusion—conditions that cirrhotic patients struggle to manage physiologically [57]. On the other hand, in liver cirrhosis, there is arterial dilation, and patients experience a hyperdynamic circulation with portal hypertension, which contribute to mesenteric congestion and bacterial/endotoxin translocation. In these circumstances, in patients with cirrhosis, there is an increase in proinflammatory cytokines that impair calcium transit in the heart, and this causes a depression of cardiac function through the mechanism of nitric oxide inhibiting cardiac contractility [58]. Overall, cardiac failure in patients with cirrhosis and SIRS contributes to a poor outcome.

One of the limitations of this study is the absence of calibration and reclassification analyses (e.g., Hosmer–Lemeshow test and NRI) to further quantify the incremental predictive value of the CAGIB score when compared with established models. Due to the limited sample size, these tests could not be reliably performed without risking statistical overfitting. Future studies with larger cohorts should include these metrics to better assess model calibration and comparative predictive performance.

## 5. Conclusions

In conclusion, our results have shown that sodium, chlorides, albumin, MAP values, and the CAGIB score are reliable predictors of 7-day and 28-day nosocomial mortality in patients with liver cirrhosis and SIRS. However, more prospective studies should be performed to confirm the relationship between laboratory biomarkers, vital signs, and prognostic scores and patients’ mortality due to advanced chronic liver disease with SIRS. We suggest that future research with larger cohorts should perform multivariable modelling to assess the relative predictive capacities of the different scores.

## Figures and Tables

**Table 1:** Formulas for calculation of non-invasive scores and ratios.

Calculation Formulas of Non-Invasive Scores and Ratios
MELDscore = 9.57 × ln (Cr) + 3.78 × ln (TBIL) + 11.2 × ln (INR) + 6.43
MELD-Na = MELD + 1.32 × (137-Na) − [0.033 × MELD × (137-Na)]
NLR = Neu/Ly
dNLR = Neu/(WBC − Neu)
PLR = Plt/Ly
S II = (Neu × Plt)/Ly
CAGIB = (Diabetes (yes = 1, no = 0) × 1.040) + (HCC (yes = 1, no = 0) × 0.974) + (TBIL × 0.005) − (ALB × 0.091) + (ALT × 0.001) + (Cr × 0.012) − 3.964.
CLIF-C ACLF = 10 × [0.33 × CLIF-C OF + 0.04 × age + 0.63 × ln (WBC) − 2]

Abbreviations: MELD—model for end-stage liver disease; Cr—creatinine; TBIL—total serum bilirubin; INR—international normalised ratio; NLR—neutrophil-to-lymphocyte ratio; Neu—neutrophils; Ly—lymphocytes; WBC—white blood cells; PLR—platelets-to-lymphocyte ratio; Plt—platelets; HCC—hepatocellular carcinoma; ALB—albumin; ALT—alanine aminotransferase, CLIF—European foundation for the study of chronic liver failure; OF—organ failure; ACLF—acute-on-chronic liver failure.

**Table 2:** Baseline clinical data and laboratory findings in patients with advanced chronic liver disease and SIRS.

Variables	Total Patients (*n* = 102)
Sex (m/f)	72/30
Age (years)	56.6 ± 13.86
SIRS	
SIRS 2 criteria	63 (61.76%)
SIRS 3 criteria	36 (35.29%)
SIRS 3 criteria	3 (2.94%)
Comorbidities	
Heart disease (yes/no)	17/85
HBP (yes/no)	27/75
Diabetes mellitus (yes/no)	13/89
Previous hospitalisation (yes/no)	41/61
Ascites (yes/no)	83/19
Variceal bleeding (yes/no)	19/79
Hepatic encephalopathy (yes/no)	72/30
Septic shock (yes/no)	13/89
Laboratory test	
Hgb (g/L)	94.33 ± 27.78
WBC (10^9^/L)	13.62 ± 7.47
Neutrophils	11.91 ± 10.49
Lymphocytes	1.15 ± 0.83
Monocytes	1.03 ± 0.81
Eosinophils	0.92 ± 2.67
Platelets (10^9^/L)	120.68 ± 92.75
TBIL (mmol/L)	132.44 ± 155.61
Alb (g/L)	29.19 ± 6.47
AST (U/L)	261.85 ± 874.91
ALT (U/L)	112.27 ± 478.76
ALP (U/L)	110.26 ± 49.90
GGT (U/L)	234.67 ± 359
sCr (µmol/L)	169.69 ± 135.16
BUN (mmol/L)	16.56 ± 30.74
LDH(U/L)	674.38 ± 580.28
CRP (mg/L)	61.06 ± 63.13
Pct (ng/L)	5.91 ± 15.59
Na (mmol/L)	134.91 ± 6.20
K (mmol/L)	4.23 ± 1.03
Cl (mmol/L)	99.1 ± 7.81
INR	2.14 ± 0.91
MELD score	24.68 ± 8.43
MELD-Na score	26.23 ± 7.96
ACLF (yes/no)	77/25
ACLF gr.1	14 (13.73%)
ACLF gr.2	21 (20.59%)
ACLF gr.3	(41.18%)
CLIF-C-OF score	10.99 ± 3.1
APACHE II score	21.23 ± 7.66
EVendo score	8.71 ± 4.96
S II	1731.03 ± 2710.45
CAGIB score	−6.93 ± 2.93
7-day survived/non-survived	69/33
28-day survived/non-survived	37/65

Abbreviations: SIRS—systemic inflammatory response syndrome; HBP—high blood pressure; WBC—white blood cells; TBIL—total serum bilirubin; Alb—albumin; AST—aspartate aminotransferase; ALT—alanine aminotransferase; ALP—alkaline phosphatase; GGT—γ-glutamine transferase; sCr—serum creatinine; BUN—blood urea nitrogen; LDH—lactate dehydrogenase; CRP—C-reactive protein; Pct—procalcitonin; Na—sodium, K—potassium; Cl—chlorides; INR—international normalised ratio; MELD—model for end-stage liver disease; MELD-Na—model for end-stage liver disease—sodium; ACLF—acute-on-chronic liver failure; CLIF—European foundation for the study of chronic liver failure; OF—organ failure; APACHE II—Acute Physiology and Chronic Health Evaluation II score; S II—systemic immune-inflammation index score.

**Table 3:** Laboratory findings and clinical scores: A comparison on the 7th day of survival in patients with SIRS and advanced chronic liver disease.

Variables	Survived Pts	Non-Survived Pts	*p*-Value
WBC	11.89 ± 6.84	17.1 ± 7.56	0.001
PT	22.94 ± 8.04	30.14 ± 13.59	0.007
INR	1.96 ± 0.73	2.51 ± 1.11	0.012
sCr	129.95 ± 87.99	249.14 ± 174.45	0.001
Cl	100.58 ± 6.97	95.91 ± 8.63	0.010
LDH	512.85 ± 192.83	1012.59 ± 900.47	0.004
MAP	84.28 ± 16.19	74.44 ± 20.98	0.020
sAP	114.89 ± 22.87	102.26 ± 26.01	0.019
APACHE II score	18.92 ± 6.73	25.91 ± 7.37	0.000
MELD score	22.45 ± 7.29	29.15 ± 8.86	0.000
MELD-Na score	24.17 ± 7.14	30.45 ± 7.98	0.000
CAGIB score	−6.42 ± 2.76	−7.94 ± 3.03	0.018

Abbreviations: WBC—white blood cells; PT—prothrombin time; INR—international normalised ratio; sCr—serum creatinine; Cl—chlorides; LDH—lactate dehydrogenase; MAP—mean arterial pressure; sAP—systolic arterial pressure; APACHE II—Acute Physiology and Chronic Health Evaluation II score; MELD—model for end-stage liver disease; MELD-Na—model for end-stage liver disease—sodium.

**Table 4:** Values of laboratory analysis and scores for the 28-day survived vs. non-survived groups of patients with SIRS and liver cirrhosis.

Variables	Survived Pts	Non-Survived Pts	*p*-Value
WBC	9.93 ± 6.07	15.57 ± 7.49	0.000
Neutrophils	7.68 ± 5.37	14.11 ± 11.84	0.000
Monocytes	0.76 ± 0.54	1.15 ± 0.88	0.009
INR	1.88 ± 0.54	2.29 ± 1.02	0.009
sCr	94.48 ± 51.65	210.84 ± 148.71	0.000
Albumin	31.65 ± 5.80	27.84 ± 6.49	0.004
Na	137.05 ± 5.25	133.56 ± 6.23	0.004
Cl	102.45 ± 6.76	97.09 ± 7.70	0.001
LDH	509.52 ± 187.27	765.12 ± 694.07	0.007
CRP	39.29 ± 40.28	73.01 ± 70.23	0.003
MAP	86.49 ± 16.41	77.82 ± 18.86	0.019
sAP	117.37 ± 20.93	106.77 ± 25.70	0.028
dAP	71.06 ± 14.83	63.34 ± 16.79	0.020
APACHE II score	17.14 ± 6.55	23.36 ± 7.43	0.000
MELD score	20.37 ± 6.78	27.24 ± 8.05	0.000
MELD-Na score	21.80 ± 6.37	28.91 ± 7.31	0.000
CAGIB score	−6.03 ± 2.58	−7.40 ± 3.03	0.021
Blood oxygen saturation	95.81 ± 3.33	90.50 ± 13.17	0.000

Abbreviations: WBC—white blood cells; INR—international normalised ratio; sCr—serum creatinine; Na—sodium; Cl—chlorides; LDH—lactate dehydrogenase; CRP—C-reactive protein; MAP—mean arterial pressure; sAP—systolic arterial pressure; dAP—diastolic arterial pressure; APACHE II—Acute Physiology and Chronic Health Evaluation II score; MELD—model for end-stage liver disease; MELD-Na—model for end-stage liver disease—sodium.

**Table 5:** Correlation between heart disease and 7-day survival.

Variable	B	S.E.	Wald	Df	Sig.	Exp (B)	95% C.I. for EXP (B)	
							Lower	Upper
Heart disease	2.064	0.722	8.161	1	0.004	7.875	1.911	32.444
Constant	−0.118	0.486	0.59	1	0.808	0.889		

Note: In the Cox proportional hazards regression, Exp (B) indicates the hazard ratio (HR) for each covariate.

**Table 6:** Correlation between GI bleeding and 7-day survival.

Variable	B	S.E.	Wald	Df	Sig.	Exp (B)	95% C.I. for EXP (B)	
							Lower	Upper
GI bleeding	0.934	0.480	3.796	1	0.051	2.545	0.994	6.516
Constant	0.000	0.408	0.000	1	1.000	1.000		

Note: In the Cox proportional hazards regression, Exp (B) indicates the hazard ratio (HR) for each covariate.

## Data Availability

All data are available from corresponding authors upon request.
